# The Quality of Life of and Social Determinants Affecting Menopausal Women in Aseer’s Healthy Cities in Saudi Arabia: A Cross-Sectional Study

**DOI:** 10.7759/cureus.31942

**Published:** 2022-11-27

**Authors:** Shahad Barkoot, Abdullah Saeed, Metrek AlMetrek, Sawsan AlShahrani, Hind AِlHomedi, Aishah AlShahrani, Ohoud AlQahtani, Shuroq AlShehri

**Affiliations:** 1 Family Medicine, Ministry of Health Holdings, Abha, SAU; 2 Action Research, Ministry of Health Holdings, Abha, SAU; 3 Nursing, Ministry of Health Holdings, Abha, SAU; 4 Health Informatics, Ministry of Health Holdings, Abha, SAU

**Keywords:** healthy cities, saudi arabia, menopausal, social determinants, quality of life

## Abstract

Introduction

Menopause is an important period in a woman’s life. It is the permanent cessation of menses for 12 months or more. Menopause can develop over a long period of time. The average age for menopause to start is 52 years, but it can begin at any time from age 40 to 58 years. Many symptoms are related to postmenopausal syndrome: hot flashes, irritability, mood swings, insomnia, dry vagina, difficulty concentrating, mental confusion, stress incontinence, urge incontinence, osteoporotic symptoms, depression, headache, and vasomotor symptoms. Quality of life (QOL) has been defined by the World Health Organization (WHO) as an “individual’s perceptions of their position in life in the context of the cultural and value systems in which they live and in relation to their goals, expectations, standards, and concerns.”

Methods

A cross-sectional study was conducted among participants from Aseer’s Healthy Cities. The sample was calculated using the formula of Swinscow and Cohen, and a total of 823 main cities were the target sample size. The sample was simply picked at random from October to December 2021. The Menopause Rating Scale (MRS)-related questionnaire is used to evaluate the symptoms of menopause in people who answer the questions themselves.

Result

The study included 869 participants, 98.3% of which are Saudi nationals. Of the participants, 82.1% were married. As regards the lifestyle of the participants in the study, 69% live with a husband and children. The mean age of the study participants was 42.5 (standard deviation (SD): 8.883) years. The mean number of participants with somatic vasomotor symptoms was 0.74 (SD: 94). The majority of the participants have sleep issues such as difficulty falling asleep, difficulty sleeping through the night, and waking up early (mean: 0.97, SD: 1.3), followed by hot flashes and sweating (mean: 0.65, SD: 1.165), and heart discomfort (mean: 0.63, SD: 12). Psychosocial symptoms are also common (mean: 0.95, SD: 1.16); the majority have depressive moods (mean: 1.09, SD: 1.35), followed by irritability (mean: 0.93, SD: 1.2), and anxiety (mean: 0.84, SD: 1.22). Physical symptoms are also prevalent, with a mean and SD of 0.91 and 1.03, respectively, with bladder problems having a mean and SD of 0.55 and 1.017, respectively. Finally, there were also sexual symptoms (mean: 0.70, SD: 1.09), with sexual problems having a mean and SD of 0.81 and 1.28, respectively.

Conclusion

The study found a high percentage of unawareness about menopause and a need to improve healthcare access and symptomatic treatment. The regression model of psychosocial risk factors is found to have a significant association with the increase of symptoms and using other medications for any reason, menstrual cycle pattern, and marital status.

## Introduction

Menopause is an important period in a woman’s life. It is the permanent cessation of menstruation for a period of at least one year [1]. Menopause can develop over a long period of time. Menopause typically starts at the age of 52; however, it can start at any age between 40 and 58 years [2]. The most common factor affecting menopause is age. With aging, the ovaries gradually lose their capacity to ovulate and generate hormones. Various operations and medical procedures might trigger menopause, including ovary removal, chemotherapy, and pelvic radiation therapy. In a premenopausal woman, a hysterectomy without the removal of the ovaries prevents menstruation but does not prevent the hormonal changes associated with menopause [3]. The menopausal transition, also known as “perimenopause,” is a predetermined period of time that starts when menstrual cycles begin to become erratic and ends when the last menstrual period occurs. Menstrual abnormalities, heavy, protracted periods interspersed with amenorrheic episodes, diminished fertility, vasomotor symptoms, and sleeplessness define this time period. Some of these symptoms may start to appear four years before menstruation stops. Estrogen levels drop as menopause approaches, whereas follicle-stimulating hormone (FSH) and luteinizing hormone (LH) levels rise. In addition to not having periods, changing cycle lengths and missed periods are indicators of the menopausal transition [4]. Furthermore, postmenopausal syndrome has a wide range of symptoms, including hot flashes, mood swings, irritability, insomnia, dry vagina, difficulty concentrating, mental confusion, stress incontinence, urge incontinence, osteoporotic symptoms, depression, headache, and vasomotor symptoms [5].

The World Health Organization (WHO) defines quality of life (QOL) as an “individual’s judgments of their place in life in relation to their goals, expectations, standards, and concerns and in the context of the cultural and value systems in which they live” [6]. Both objective and subjective indicators are included. The subjective measure of quality of life is the level of pleasure or satisfaction experienced as a result of how one perceives and assesses their life. This includes aspects such as the welfare state, as well as economic, political, cultural, and environmental concerns [7]. Women going through menopause have lower QOL [8]. Approximately 3,000 women had their health-related quality of life assessed as part of the Study of Women’s Health Across the Nation (SWAN), which discovered that numerous menopausal symptoms were also linked to a lower quality of life [9]. Regarding menopausal status, a study conducted in Belgrade, Serbia, and published in 2020 evaluated the factors associated with mental health quality of life (QOL), such as depressed mood, anxiety, and poor memory and sleep, among midlife women. The results showed that in women who are in the premenopausal period, lower household monthly income was associated with a higher level of depressive mood, higher body mass index was associated with higher anxiety, and having gynecological illnesses and menopause-related symptoms were associated with poorer memory and that in women who are in the postmenopausal period, those who lived on the outskirts of cities, were single, and held sedentary jobs had worse depression. Moreover, those with lower education levels had more anxiety, those with lower education levels and menopause-related symptoms had a poor memory, and those who did not participate in regular recreation had better sleep. It was determined that not only menopausal symptoms needed to be addressed to improve QOL of pre- and postmenopausal women. When they are reaching midlife, women should also receive advice on behavioral and personal adjustments [10].

To evaluate the quality of life (QOL) of postmenopausal women in the rural areas of Puducherry, India, and identify the contributing factors that led to the prevalence of one or more symptoms in the vasomotor (23.8%), psychological (87%), and sexual (68%) domains, a community-based cross-sectional study was carried out. Socioeconomic status was discovered to be related to both vasomotor and psychological symptoms. Additionally, there was a link between age and psychological symptoms. This study came to the conclusion that the QOL of postmenopausal women was negatively impacted by menopause-related symptoms [11].

A cross-sectional research of 542 postmenopausal Saudi women in Riyadh, Saudi Arabia, found that vasomotor, psychosocial, physical, and sexual problems affected 41% (n = 224), 14.4% (n = 78), 57% (n = 307), and 12.7% (n = 69) of the women severely or moderately, respectively. Lack of emotional support was linked to severe/moderate vasomotor (adjusted odds ratio (aOR): 1.5, 95% confidence interval (CI): 1.1, 2.3), psychosocial (aOR: 2.0, 95% CI: 1.2, 3.4), and physical (aOR: 1.7, 95% CI: 1.2, 2.6) symptoms, according to multivariate logistic regression. Severe/moderate sexual symptoms were related to a lack of concrete social support (aOR: 1.9, 95% CI: 1.0, 3.4). Women were more likely to experience moderate or severe menopausal symptoms if they worked (aOR: 1.8, 95% CI: 1.1, 3.2), were obese (aOR: 2.0, 95% CI: 1.0, 4.1), rented housing (aOR: 3.9, 95% CI: 1.2, 13.1), or had a retired spouse (aOR: 1.6, 95% CI: 1.0, 2.4). They also came to the conclusion that creating programs for postmenopausal women, their spouses, and other family members could enhance social support and, consequently, the quality of life of postmenopausal women [12].

In Riyadh, Saudi Arabia, during October and November 2010, a cross-sectional study was carried out to ascertain the prevalence and severity of menopausal symptoms and their effect on the quality of life among Saudi women visiting primary care facilities. The findings revealed that joint and muscular pain (80.7%), physical and mental tiredness (64.7%), and hot flashes and sweating (47.1%) were the symptoms that were most frequently mentioned. Compared to other categories, somatic and psychological complaints were significantly more common among perimenopausal women. Perimenopausal women had a higher mean overall quality of life score, despite the fact that the total Menopause Rating Scale (MRS) score (MRS 9) showed that the symptoms were minor in intensity. According to the findings of this study, Saudi women had MRS scores that indicated reduced symptom severity, higher quality of life, and the ability to manage climacteric symptoms [13].

A cross-sectional study revealed that in 228 women in the city of Abha on November 5, 2016, the majority of women expressed difficulty with their joints and muscles (96.1%), as well as irritation (94.7%), anxiety (89%), and hot flashes and sweating (80.7%). The mean overall score of the Menopause Rating Scale (MRS) was 15.25 (SD: 6.01). The mean score for somatic symptoms was 6.36 (SD: 3.01), for psychological symptoms was 6.05 (SD: 2.54), and for urogenital symptoms was 2.84 (SD: 2.25). Higher MRS and poor quality of life were substantially correlated with marital status, lower educational attainment, parity, lack of exercise, and chronic disease status. According to the findings of this study, women in Abha, Saudi Arabia, had moderate MRS scores, which indicate a moderately poor quality of life and some coping skills [14].

A woman’s menopause is a crucial time in her life since it not only signifies the end of her ability to conceive children but is also accompanied by a number of physical, vasomotor, psychological, and sexual issues. Knowing the age of natural menopause is crucial because as you age, your risk of developing heart disease, osteoporosis, endometrial cancer, and breast cancer increases [15]. The quality of life at this time is very important to both women and their treating physicians because women are projected to live a quarter to a third of their lives following menopause [16]. Menopausal symptoms have a strong negative impact on QOL [17].

The objective of this study was to bring it all up to date. The QOL of menopausal women in Arab and Gulf nations is poorly understood. Hence, this study aims to evaluate the quality of life of menopausal women and the social variables affecting them in Aseer’s Healthy Cities in Saudi Arabia.

## Materials and methods

Methodology

A cross-sectional study was conducted that included female participants who are at least 40 years old, both citizens and residents. According to the formula of Swinscow and Cohen, the sample size for the primary target cities should be 823, distributed and chosen at random.

Timeframe

The sample was simply picked at random from October to December 2021.

Data collection

Menopausal symptoms were evaluated using a questionnaire comparable to the Menopause Rating Scale (MRS), a self-administered tool that has been extensively validated and used to assess the severity of menopausal symptoms in several clinical and epidemiological investigations, as well as in research on the causes of menopausal symptoms [10]. The scale was created and standardized as a self-administered scale to evaluate the severity of symptoms over time, analyze pre- and postmenopausal replacement therapy changes, and assess symptoms and complaints of aging women under varied settings. The scale, which is offered in 10 languages, has gained widespread use. The scale consists of two parts. The first section comprises sociodemographic information including 13 questions about age, marital status, parity, number of abortions, number of gravidities (pregnancies) if any, menstrual status, age at which menstruation stopped (if it did), educational level, employment status, smoking status, use of hormone replacement therapy, calcium intake, and whether or not the participant has had a hysterectomy with bilateral oophorectomy [18]. The second section consists of 11 questions on the physical, mental, and urogenital symptoms of menopause, including hot flashes, sweating, heart discomfort, sleep issues, sad mood, irritability, anxiety, physical tiredness, bladder issues, dry vagina, and joint and muscular soreness [19]. A random sample of 45- to 65-year-old women from the German Cohort Study of Women’s Health underwent two applications of the MRS, with a 14-day gap between each measurement, and the sample’s response rate was 70%. The sum score of the two assessments has a correlation coefficient (or Pearson’s correlation coefficient) of 0.82. The great repeatability of the test results highlights the good practical applicability and supports our clinical expertise. The MRS is a dependable scale that can be used in clinical settings to measure and continuously monitor menopausal complaint dynamics [20]. The questionnaire was translated into simple Arabic, and we filled it out using a firsthand observation interview.

Statistical analysis

The Statistical Package for the Social Sciences (SPSS) version 21 software (IBM SPSS Statistics, Armonk, NY, USA) was used to gather, code, and statistically analyze data. Categorical variables were presented as frequencies and percentages, and descriptive statistics were used. For continuous variables, measures of central tendency and measures of dispersion were computed. The chi-square test was used in analytical statistics to check for associations and/or differences between categorical variables. If the frequency in at least one cell is less than five, Fischer’s exact test is used in place of the chi-square test.

## Results

There were a total of 869 participants, 98.3% of whom are Saudi nationals and 1.7% are non-Saudi nationals. Of the participants, 82.1% were married, 6% were single, 7% were divorced, and 4.9% were widows. Moreover, 69% lived with a husband and children, 10% with a husband only, 8% alone, 8.7% with children, 2.4% with parents, and 1.1% with other relatives. Regarding home ownership, only 23.2% of the participants rented their homes, while 76.9% were homeowners. Regarding the type of construction of their houses, 9.4% were typical local buildings, while 90.6% were new concrete and aluminum homes. For walking areas, 37.7% have backyards, 18.1% have gardens, 11.7% have public walk areas, and 1.5% have vacant lots; also, 31.1% have gym memberships. As regards income, with a cut point of 8,000 Saudi riyals (SAR), 44.2% earn <8,000 SAR, while 55.8% earn >8,000 SAR. Regarding how people behave when spending money, the study found that 43.2% spend >8,000 SAR, while 56.8% spend <8,000 SAR. The percentage of those without a high school diploma is 6.2%, 7.1% can write, 4.8% obtained primary education, 21.4% obtained intermediate education, 56.8% obtained a secondary school diploma, and 3.7% were at university level. Furthermore, 1.2% were working, and 98.8% were not working. Most of the participants used drugs of any kind (64.3%). Operations in the female reproductive system were done in 14.4% of the participants. Only 30.3% of women exercised every day, and the most popular form of exercise was walking, followed by strenuous exercise, and running. The percentage of people who had some knowledge of the symptoms associated with the menopausal stage was 96.4%, but only 5.2% had used nonmedical methods to alleviate them. Only 14.5% had gone to a primary healthcare facility or hospital for treatment or education, and only 15.3% had talked to a doctor about getting checked out early and staying healthy (Table 1).

**Table 1:** Frequency and percentage of study variables (N = 869) SAR: Saudi riyals

Variable	Percentage	Frequency
Nationality		
Saudi	98.3	869
Non-Saudi	1.7	15
Marital status		
Single	6	53
Married	82.1	726
Divorced	7	62
Widow	4.9	43
With whom do you live?		
With husband and children	69.1	611
With husband	10.5	93
Alone	8.1	72
With children	8.7	77
With parent	2.4	21
With other relatives	1.1	10
Residence ownership		
Own	76.8	679
Rent	23.2	205
Type of construction		
New	90.6	801
Traditional	9.4	83
Walking area		
Backyard	37.7	333
Garden	18.1	160
Walk area	11.7	103
Gym	31.1	275
Other	1.5	13
Income		
<8,000 SAR	44.2	391
>8,000 SAR	55.8	493
Expenditure		
<8,000 SAR	56.8	502
>8,000 SAR	43.2	382
Educational level		
Illiterate	6.2	55
Write	7.1	63
Primary	4.8	42
Intermediate	21.4	189
Secondary	56.8	502
University	3.7	33
Job title		
Working	1.2	11
Not working	98.8	873
Do you use any kind of drugs?		
Yes	64.3	568
No	35.7	286
Operations in the female reproductive system		
Yes	14.4	127
No	85.6	757
Do you exercise daily?		
Yes	30.3	268
No	69.7	616
Type of exercise		
Run	1.4	12
Walk	36	318
Swim	0.5	4
Intense	3.3	29
Other	0.7	6
Dance	0.5	4
Cardio	0.8	7
Do you have knowledge of the symptoms of the stage of regeneration (formerly called menopause)?		
Yes	3.6	32
No	96.4	852
Do you use nonmedical alternatives to relieve the symptoms of this stage?		
Yes	5.2	46
No	94.8	838
Do you use preventive or curative medications for the symptoms?		
Yes	14.5	128
No	85.5	756
Did you ever, during your visit to primary healthcare centers or hospitals, receive medical advice or health education about the stage of regeneration (formerly menopause), its symptoms, and the proposed treatment options for it?		
Yes	7.1	63
No	92.9	821
Did you ever, during your visit to primary healthcare centers or hospitals, receive medical advice about early examination of diseases, periodic annual examination, or health promotion?		
Yes	15.3	135
No	84.7	749

The mean age of the study participants was 42.5 (SD: 8.8) years. The mean duration of smoking is 11.2 (SD: 8.5) years. The mean number of participants with somatic vasomotor symptoms was 0.74 (SD: 94). The majority of the participants have sleep issues such as difficulty falling asleep, difficulty sleeping through the night, and waking up early (mean: 0.97, SD: 1.3), followed by hot flashes and sweating (mean: 0.65, SD: 1.165), and heart discomfort (mean: 0.63, SD: 12). Psychosocial symptoms are also common (mean: 0.95, SD: 1.16). The majority have depressive moods (mean: 1.09, SD: 1.35), followed by irritability (mean: 0.93, SD: 1.2), and anxiety (mean: 0.84, SD: 1.22). Physical symptoms are also prevalent, with a mean and SD of 0.91 and 1.03, respectively, with bladder problems having a mean and SD of 0.55 and 1.017, respectively. Finally, there were also sexual symptoms (mean: 0.70, SD: 1.09), with sexual problems having a mean and SD of 0.81 and 1.28, respectively (Table 2).

**Table 2:** Mean score for menopausal status and factors

Variable	Mean	Standard deviation
Age in years	42.56	8.883
Duration of smoking in years	11.214	8.4649
Duration of daily exercises	1.39	.488
Hot flashes and sweating (sudden sensation of heat and intense sweating)	0.65	1.165
Heart discomfort (unusual awareness of heartbeat, heart skipping, heart racing, and tightness)	0.63	1.120
Sleep problems (difficulty in falling asleep, difficulty in sleeping through the night, and waking up early)	0.97	1.302
Depressive mood (feeling down, sad, on the verge of tears, lack of drive, and mood swings)	1.09	1.351
Irritability (feeling nervous, inner tension, and feeling aggressive)	0.93	1.297
Anxiety (inner restlessness and feeling panicky)	0.84	1.228
Sexual problems (change in sexual desire, sexual activity, and satisfaction)	0.81	1.281
Bladder problems (difficulty in urinating, increased need to urinate, and bladder incontinence)	0.55	1.017
Dryness of vagina (sensation of dryness or burning in the vagina and difficulty with sexual intercourse)	0.61	1.127
Vasomotor	0.74	0.94
Sexual	0.70	1.09
Psychosocial	0.95	1.16
Physical	0.91	1.03

Using a linear regression model for all sociodemographic variables, with psychosocial symptoms as outcomes and other variables as predictors and significance set at B value of less than 0.05, the use of any medication, menstrual cycle disturbance, and marital status are considered risk factors for increased psychosocial symptoms in menopausal women (Table 3).

**Table 3:** Linear regression model for predictive variable

Linear model	B value	Standard error	Beta	Test statistic	Significant
(Constant)	1.563	0.306		5.101	0.000
Do you use any drugs?	-0.256	0.108	-0.107	-2.374	0.018
Menstrual cycle nature	0.152	0.067	0.103	2.275	0.023
Marital status	-0.215	0.107	-0.090	-2.017	0.044

## Discussion

Postmenopausal women’s family demands vary as a result of challenges in their lives. They require extra help from their family. Negative attitudes such as loneliness, malaise, and family troubles will emerge if they do not receive help. Individuals’ ability to cope with stress is increased, and psychological and physical symptoms are reduced when they receive social support in the form of emotional and informational support. According to studies, social support has a negative and meaningful relationship with the experiences of women going through menopause, and increasing social support from various sources reduces physical and mental problems. The husband is the most important and closest person who can support a woman in this challenge with a correct understanding of her situation and problems [21]. According to research, the average social support perceived by women after implementing an educational program for husbands was greater than before, and the difference was significant. To put it in another way, if the husband has more information about his wife’s emotional and physical health, he will be able to better understand and assist her. Social support improves the physical and mental health of women and helps them avoid depression [22]. Women with lower levels of education had more severe symptoms, whereas women with greater levels of education were better aware of menopausal symptoms and strategies for dealing with them, as well as more inclined to seek treatment for their symptoms. In addition, educated women reach menopause at a younger age than illiterate women. An increasing amount of evidence suggests that women who stay at home have a lower quality of life and more menopausal symptoms than those who work. Women who are financially stable have a better quality of life during menopause. This is because they have easier access to healthcare and counseling to deal with menopausal symptoms [23].

According to the findings of our study, multiparity and many births can result in physical and psychological problems, lowering the QOL. Parental stress and responsibility, as well as financial concerns, may increase as the number of children increases. Mothers’ childcare concerns, namely, providing a nice life for their children and paying attention to their comfort, always take precedence over their personal convenience, which correlates with their quality of life. Since there was a strong negative link between QOL and the number of stillbirths, it was also possible to figure out that the chances of having a stillborn child increased with each pregnancy [24].

Recommendations

Menopausal symptoms are fairly prevalent and should not be disregarded, as evidenced by the relatively low quality of life brought on by menopausal symptoms that were seen in this study. Better care must be made available to women going through this stage of life to improve their quality of life. To focus on menopausal women and their needs, it is proposed that menopausal clinics be developed within the present primary healthcare system. The Leonardo project of “care managers,” which acts as a link between general practitioners (GPs), patients, and other medical specialists for the treatment of chronic diseases, may be a good substitute for the standard care given to perimenopausal women as they navigate this challenging stage of life. We also advise encouraging education and a better way of life at the local level since women who are more aware, interested, and empowered engage with healthcare professionals more effectively and work to take steps that will lead to healthier results. We advise future research to employ more clinic visits, thorough interviews, or focus groups instead of the single computerized self-reporting survey that was used in this one.

## Conclusions

In contrast to past research conducted in Saudi Arabia, our study unequivocally shows that women in Abha, Saudi Arabia, have a somewhat poor quality of life, as evidenced by higher MRS scores. Somatic symptoms, notably pain in the muscles and joints, are the most often reported symptoms, and menstrual cycle type and medication use have a significant impact on psychosocial symptoms. Widower status, parity, low education, inactivity, and unemployment are factors that have a major impact on menopausal symptoms, whereas an improved quality of life is linked to greater levels of education and a healthy lifestyle that includes regular exercise 3-5 times per week.
